# Poisson-Gamma Mixture Spatially Varying Coefficient Modeling of Small-Area Intestinal Parasites Infection

**DOI:** 10.3390/ijerph16030339

**Published:** 2019-01-26

**Authors:** Frank Badu Osei, Alfred Stein, Anthony Ofosu

**Affiliations:** 1 Faculty of Geo-Information Science and Earth Observation (ITC), University of Twente, 7522 NB Enschede, The Netherlands; a.stein@utwente.nl; 2 Policy, Planning, Monitoring and Evaluation (PPME)–Ghana Health Service; Accra, Ghana; anthony.ofosu@ghsmail.org

**Keywords:** intestinal parasites, spatio-temporal, Poisson-gamma, Bayesian, integrated nested Laplace approximations

## Abstract

Understanding the spatially varying effects of demographic factors on the spatio-temporal variation of intestinal parasites infections is important for public health intervention and monitoring. This paper presents a hierarchical Bayesian spatially varying coefficient model to evaluate the effects demographic factors on intestinal parasites morbidities in Ghana. The modeling relied on morbidity data collected by the District Health Information Management Systems. We developed Poisson and Poisson-gamma spatially varying coefficient models. We used the demographic factors, unsafe drinking water, unsafe toilet, and unsafe liquid waste disposal as model covariates. The models were fitted using the integrated nested Laplace approximations (INLA). The overall risk of intestinal parasites infection was estimated to be 10.9 per 100 people with a wide spatial variation in the district-specific posterior risk estimates. Substantial spatial variation of increasing multiplicative effects of unsafe drinking water, unsafe toilet, and unsafe liquid waste disposal occurs on the variation of intestinal parasites risk. The structured residual spatial variation widely dominates the unstructured component, suggesting that the unaccounted-for risk factors are spatially continuous in nature. The study concludes that both the spatial distribution of the posterior risk and the associated exceedance probability maps are essential for monitoring and control of intestinal parasites.

## 1. Background

The common intestinal parasites, *Ascaris lumbricoides* (roundworms), *Trichuris trichiura* (whipworms), and *Necator americanus* or *Ancylosttoma duodenale* (hookworms) are estimated to infect more than 1 billion people [1,2]. Infection occurs mainly by means of contact with infected environments, hand-to-hand contact, or contaminated food or water (fecal-oral) [3]. Intestinal parasites thrive under climatic and environmental conditions such as high temperatures, severe precipitation, and adequate soil moisture [4,5,6]. Without effective treatment, intestinal parasites infection can lead to blood loss and development of iron deficiency anemia. In children, infections can retard growth, cause anemia, and create cognitive and physical deficiencies [7,8,9]. While chemotherapy has been adopted as effective for reducing the burden of intestinal parasites, it is unfeasible without preventive measures targeted at the demographic risk factors. Alternative interventions using the best available evidence is therefore crucial. In this regard, development of statistical models to associate and explain the spatial variation of intestinal parasites risk can establish baseline demographic factors to target.

Resource-poor countries in Asia, sub-Sharan Africa, and Latin America have the highest prevalence and considerable burdens due to limited access to safe water supply and poor sanitation [1,2]. Ghana is among the sub-Saharan countries with a high prevalence of intestinal parasites infections [1,2]. The disease has constantly been listed amongst the top five outpatient morbidities. Prevalence has been reported to be between 2% and 78% for various parasites and specific population groups [10,11,12,13,14,15]. Amongst food vendors in the Accra Metropolitan area, prevalence has been estimated to be 21.6% [16]. Amongst school children in the Kintampo North Municipality, the prevalence of hookworm infection was estimated to be 39.1%, with significant risk factors being age, malaria parasitemia, lack of health care, school area, levels of antibodies against hookworm, and low consumption of animal foods [15]. Asymptomatic carriage among psychiatric patients was estimated to be 13.5% among some Ghanaian orphanages [13]. In some parts of Ghana, the prevalence of hookworms has been associated with sociodemographic conditions such as age, malaria parasitemia, lack of health care, and low consumption of animal foods [15].

Globally and historically, the emphasis has largely been based on biological characteristics of the parasites and the developments of treatments and vaccines, whereas less emphasis has been made to explain the spatial and temporal patterns. Small-area modelling of infection risk is important for public health assessment of risk factors and their effects. This is also useful for developing site-specific interventions that are especially essential in resource-poor countries [6,17,18]. The recommendation by the World Health Organization (WHO) is to ensure periodic administration of albendazole and mebendazole to at-risk populations [1,19]. In order to ensure effective administration to at-risk populations and other interventions, we need to deepen our knowledge on the effects of demographic risk factors on the spatial distribution of infections. Thus, understanding the effects of risk factors on the spatial distribution of infection risk is important for monitoring and control. Several studies have associated infections with climatic and environmental conditions such as high temperatures, severe precipitation, and adequate soil moisture [6,20]. Likewise, infections have often been associated with sociodemographic conditions such as poverty, poor sanitation, and poor drinking water [21,22,23]. In Ghana, studies have mostly focused on either the biological or anthropogenic characteristics of the individuals affected. Besides, prevalence and associations have either been estimated for single geographic units or among specific population categories. Hence, they are unable to evaluate the effect of demographic variables on the spatial patterns of infection. Some prevalence studies have used exploratory methods to derive associations with environmental and demographic factors [24,25,26]. While these studies suggest the uneven distribution of risk, they are unable to evaluate the spatial effects of risk factors.

In our previous study [27], we found evidence of spatial clustering, suggesting that spatially continuous factors like unplanned urbanization, unsafe drinking water, and unsafe toilets could modulate the occurrences of intestinal parasites infections. Our objective in this study is to associate the spatial distribution of the risk of infection counts with these demographic variables. The hierarchical Bayesian modeling framework offers a flexible and robust platform to account for spatial clustering, temporal random effects, and spatio-temporal interaction effects. Given the complexity of the relationships with demographic factors, non-stationarity in the impacts of demographic factors is foreseeable. We thus anticipate that the effects of the demographic variables on infections will vary across space, a phenomenon rarely accounted for in most spatial epidemiologic models. Also, over-dispersion may occur due to outlying counts. Given the above complexities, the study presents a Poisson-gamma mixture varying coefficient model that accounts for both the over-dispersion and the varying coefficients effects. Markov chain Monte Carlo (MCMC) simulation is a commonly used method for estimation and inference in Bayesian models. We used the integrated nested Laplace approximation (INLA) for approximate Bayesian inferences [28,29] due to its fast computational time over the MCMC. 

## 2. Study Area and Data

**Figure 1:**
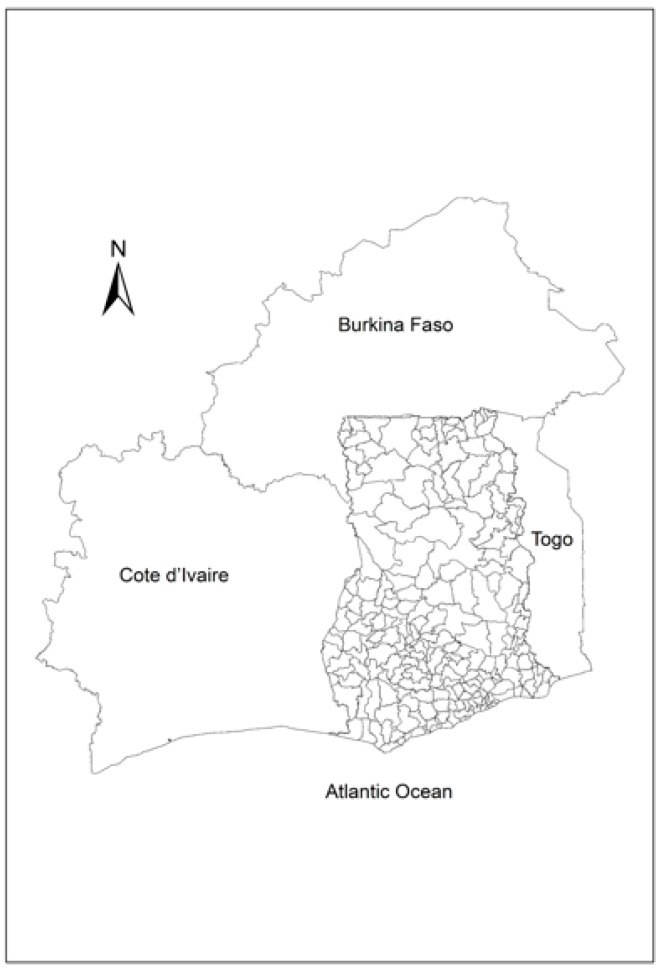
District map of Ghana showing its neighboring countries; Cote d’Ivoire (left), Burkina Faso (top) and Togo (right). This map was created using ArcGIS software (version 10.1, ESRI Inc. Redlands, CA, USA. https://www.esri.com/).

Ghana is a developing country on the west coast of Africa (Figure 1). The country has a land area of 238,589 km^2^ with a population of approximately 27 million and subdivided into 216 administrative districts. Our analysis, however, was restricted to the 170 districts for which data had been recorded. We obtained yearly intestinal parasites morbidity records of outpatient departments (OPD) from 2010 to 2014 from the Centre for Health Information and Management (CHIM) of the Ghana Health Services (GHS). Population projections per district per year from 2010 to 2014 were obtained from the Ghana Statistical Service.

Increasing access to safe water and sanitation is a successful approach to reduce the menace of intestinal parasites as supposed by previous studies [21,22,23]. Ghana is transitioning to a development where access to safe water and sanitation have been compromised by population growth. Coupled with population growth and unplanned urbanization, poor living conditions emerge. This is associated with increased rural–urban migration, lack of or inadequate safe water supply, and sanitation, all of which plausibly combine to increase the risk of intestinal parasites. Although some improvements of water and sanitation have been made [30], the coverage is still only 60% [31]. Therefore, it is conceivable to expect that the risk of intestinal parasites infection increases with increasing proportions of inhabitants with unsafe water, unsafe toilets, and unsafe liquid waste disposal. From the 2010 Ghana Population and Housing Census data, we estimated unsafe drinking water (wat) as the percentage of the district’s population that do not have access to pipe-borne water (either in dwellings, outside dwellings, or public standpipes). We estimated unsafe toilet (toi) as the percentage of the district’s population without access to flush toilet and unsafe liquid waste disposal (disp) as the percentage of the district’s population that disposes their liquid waste either on the streets or on the compound.

## 3. Methods

In this study, we developed a spatio-temporal Poisson-gamma mixture spatially varying model to associate intestinal parasites counts with demographic variables. To do so, we considered the double $\left\{y_{it},n_{it}\right\}$ representing the spatio-temporal outcomes of intestinal parasites counts and population data disaggregated by districts i=1,…,m=170 and temporal periods t=1,…,T=5. Such sampling models are typically realizations from the Poisson process $y_{it}|\varsigma_{it}=Poi\left(n_{it}\varsigma_{it}\right)$ with likelihood $f\left(y_{it}\right)=exp\left(y_{it}log\left(n_{it}\varsigma_{it}\right)-n_{it}\varsigma_{it}-log\left(y_{it}!\right)\right)$, where $\varsigma_{it}$ is an unknown risk and $n_{it}$ is an offset to control population differentials. The Poisson distribution is parameterized by $n_{it}\varsigma_{it}$ being both the mean and variance, thus $E\left(y_{it}\right)=V\left(y_{it}\right)=n_{it}\varsigma_{it}$. This assumption is unrealistic and often leads to over-dispersion, i.e., where $E\left(y_{it}\right)<V\left(y_{it}\right)$. To avoid this situation, we express $y_{it}|\varsigma_{it}=Poi\left(\left(n_{it}\varsigma_{it}\right)\alpha \right)$ by introducing an extra gamma noise parameter $\alpha \sim gamma\left(\vartheta_{1},\;\vartheta_{2}\right)$, also called the dispersion parameter, such that $V\left(y_{t}\right)=n_{it}\varsigma_{it}+{\left(n_{it}\varsigma_{it}\right)}^{2}/\alpha$. This can be factorized into the over-dispersion component α and the linear component $log(\eta_{it})=\log\left(n_{it}\varsigma_{it}\right)$. Note that integration over the conjugate random effect term α leads to the Poisson-gamma mixture density, also called the negative-binomial density.

(1) $$f\left(y_{it}\right)=\frac{\Gamma \left(y_{it}+\vartheta_{1}\right)}{y_{it}!\Gamma \left(\vartheta_{1}\right)}{\left(\frac{\vartheta_{2}}{\vartheta_{2}+1}\right)}^{\vartheta_{1}}{\left(\frac{1}{\vartheta_{1}+1}\right)}^{y_{it}}$$

This can be written as $y_{it}|\varsigma_{it}=NegBin\left(\vartheta ,n_{it}\varsigma_{it}\vartheta \right)$. As the dispersion parameter increases, the variance converges to the same value as the mean, and the Poison-gamma turns into a Poisson distribution. Our interest lies in the demographic, spatial, temporal, and spatio-temporal variation in the risk $\varsigma_{it}$. The assumption of stationarity in neighborhood demographic effects is difficult to meet because of differences in neighborhood specific characteristic and unobserved factors that can locally influence the disease outcomes. To remedy these concerns, we allowed the neighborhood demographic effects to vary by location. As we have previously described in our study of a similar setting [32], we expressed the log of the risk as a linear combination of spatially varying effects of the demographic variables, spatial random effects, temporal effects, and space-time random effects:

(2) $$log\varsigma_{it}=\eta_{it}=\beta_{0}+v_{i}+u_{i}+\gamma_{t}+\varphi_{it}+\beta_{it}^{wat}wat_{it}+\beta_{it}^{toi}toi_{it}+\beta_{it}^{disp}disp_{it}$$

Here, $\beta_{0}$ denotes the overall level of the risk on a log scale. The variables $wat_{it}$, $toi_{it}$, and $disp_{it}$ are the continuous regressors at location *i* with mean effects coefficients $\beta^{wat}$, $\beta^{toi}$, and $\beta^{disp}$; $urb_{i}$ is a categorical regressor at location *i* with mean effects coefficient $\beta^{urb}$. We assumed the covariates to be static, $wat_{it}=wat_{i}$, $toi_{it}=toi_{i}$, and $disp_{it}=disp_{i}$ due to the unavailability of yearly census data. Hence, $\beta_{it}^{wat}=\beta_{i}^{wat}$, $\beta_{it}^{toi}=\beta_{i}^{toi}$, and $\beta_{it}^{disp}=\beta_{i}^{disp}$. We included $u_{i}^{wat}$, $u_{i}^{toi}$, and $u_{i}^{disp}$ as differential spatially varying effects that account for the varying effects of the continuous covariates. The parameters, $\beta_{i}^{wat}=\beta^{wat}+u_{i}^{wat}$, $\beta_{i}^{toi}=\beta^{toi}+u_{i}^{toi}$, and $\beta_{i}^{disp}=\beta^{disp}+u_{i}^{disp}$ can be viewed as spatially varying coefficients for the variables $wat_{i}$, $toi_{i}$, and $ud_{i}$, respectively.

We accommodated unmeasured confounders that induce spatial correlation by introducing a spatially correlated random effect term $u_{i}$. We specified the distribution of $u_{i}$ conditional on the set $u_{-i}=u_{i\neq j}=\left\{u_{1},\ldots ,u_{i-1},u_{i+1},\ldots ,u_{n}\right\}$ as an intrinsic conditional autoregressive (iCAR) prior [33] $u_{i}|u_{-i}\sim Normal\left({\bar{u}}_{i},\sigma_{u}^{2}/w_{i+}\right)$, where ${\bar{u}}_{i}=\sum_{j}w_{ij}u_{j}/w_{i+}$ and $w_{i+}=\sum_{j}w_{ij}$. Here $w_{ij}$ is an m×m spatial proximity matrix such that $w_{ij}=1$ if *i* and *j* are neighbors i~j (share a common boundary), and 0 otherwise. Setting $w_{ij}=0$ ensures that locations do not predict themselves. We imposed a sum to zero constraint $\sum u_{i}=0$ to ensure identifiability. The mean of the joint prior distribution $p\left(u_{i}|\sigma_{u}^{2}\right)$ is therefore set to zero with precision matrix $\sigma_{u}^{-2}Q_{u}$, where $Q_{u}$ is a structural matrix deduced from the binary proximity matrix $w_{ij}$. The structural matrix $Q_{u}$ has diagonal elements $Q_{ii}=\sum_{j}w_{ij}$ and off-diagonal elements $Q_{ij}=-w_{ij}$. For brevity, we write $u_{i}\sim iCAR\left(Q_{u},\sigma_{u}^{2}\right)$. We included the term $v_{i}\sim N\left(0,\sigma_{v}^{2}\right)$ to account for unobserved random effects. The convolution term $v_{i}+u_{i}$ avoids choosing between heterogeneity and clustering. This term can be interpreted as random spatial adjustments to the overall intercept, and $\beta_{0}+v_{i}+u_{i}$ is interpreted as varying intercepts across the districts. The term $\gamma_{t}$ accounts for the temporal processes and $\varphi_{it}=\left\{\varphi_{11},\ldots ,\varphi_{mT}\right\}$ are space-time random interactions. Considering the years t=1,…,T as Gaussian vectors, we specified first-order random walk processes $\gamma_{t}=\gamma_{t-1}+\Delta \gamma$, where $\Delta \gamma \sim Normal\left(0,\sigma_{\gamma }^{2}\right),$ to penalize abrupt yearly jumps. For the space-time interactions, we specified $\varphi_{it}\sim Normal\left(0,\sigma_{\varphi }^{2}\right)$. This specification of $\varphi_{it}$ is referred to as unstructured temporal and unstructured spatial effects [34]. To ensure that spatial correlations of the of the varying effects of the covariates are captured, we specified $u_{i}^{\left(\cdot \right)}=\left\{u_{i}^{wat},u_{i}^{toi},u_{i}^{disp}\right\}$ as $u_{i}^{\left(\cdot \right)}\sim iCAR\left(Q_{u},\sigma_{u^{\left(\cdot \right)}}^{2}\right).$

## 4. Bayesian Inference

We adopted the Bayesian hierarchical specification through the INLA to estimate the model parameters. INLA provides accurate estimates of the integrals through a Laplace approximation, namely a deterministic algorithm proposed by Rue and Martino [28]. The Bayesian paradigm requires calculating the posterior distribution of the unknown parameters given the data. If the vector $\psi_{1}=\left\{\varsigma ,\beta_{0},v,u,\gamma ,\varphi ,\beta^{\left(\cdot \right)},u_{i}^{\left(\cdot \right)}\right\}$ is the full Gaussian latent field and $\psi_{2}=\left\{\sigma_{v}^{2},\sigma_{u}^{2},\sigma_{\gamma }^{2},\sigma_{\varphi }^{2},\sigma_{u^{\left(\cdot \right)}}^{2}\right\}$ is a vector of hyper-parameters, then the joint posterior distribution of $\psi_{1}$ and $\psi_{2}$ given the data likelihood equals $p\left(\psi_{1},\psi_{2}|y\right)\propto p\left(y|\psi_{1},\psi_{2}\right)\cdot p\left(\psi_{1}|\psi_{2}\right)\cdot p\left(\psi_{2}\right)$. We can equate $y|\psi_{1},\psi_{2}\sim p\left(y|\psi_{1},\psi_{2}\right)$, $\psi_{1}|\psi_{2}\sim p\left(\psi_{1}|\psi_{2}\right)$ and $\psi_{2}\sim p\left(\psi_{2}\right)$ as the data, the process, and parameter models, respectively. The model parameters are assumed to be random with prior distributions assigned at each stage of the hierarchy. We assigned a non-informative low precision zero-mean Gaussian prior $\beta^{\left(\cdot \right)}\sim N\left(0,0.001\right)$ for the fixed effects $\beta^{\left(\cdot \right)}=\left\{\beta^{wat},\beta^{toi},\beta^{disp}\right\}$, and an independent diffuse prior $p\left(\beta_{0}\right)\propto constant$ for the intercept. For the precision parameters, $\tau_{l}=1/\sigma_{l}^{2}$, $l=v,\;u,\;\gamma ,\;\varphi ,u_{i}^{\left(\cdot \right)}$, which are at the lowest level of the hierarchy, we assigned a minimally informative Gamma distribution of the form $log\tau_{l}\sim logGamma\left(1,\;0.0005\right)$ to benefit from the attractive features of conjugacy.

Separate models were fitted for the Poisson and Poisson-gamma likelihoods and their deviance information criterion (DIC) values were compared. The DIC is a generalization of the Akaike’s information criterion (AIC). A small DIC value corresponds with a good predictive performance of the model. The $DIC=\bar{D}+p_{D}$ is the sum of the model fit $\bar{D}$ and model complexity $p_{D}$ [35] and is defined analogously to the Akaike information criterion (AIC) in frequentist modeling. Negative twice the log-likelihood of the deviance informs the model fit, while the effective number of parameter informs the model complexity.

Posterior means and confidence intervals of the risk $\varsigma_{it}$ and all unknown parameters (fixed and random) were estimated by drawing large samples (N=100,000) from the posterior marginals. We estimated the relative contribution of the structured and unstructured spatial effects using a fraction of the marginal variability of the structured spatial effects $\sigma_{u}^{2}$ over the total marginal variability $\sigma_{u}^{2}+\sigma_{v}^{2}$. Since the parameter $\sigma_{u}^{2}$ is not directly available, we used its empirical estimate ${\hat{\sigma }}_{u}^{2}=\sum_{i}\left(u_{i}-\bar{u}\right)/\left(n-1\right)$. The relative contribution of spatially structured effects is thus given by $frac_{u}={\hat{\sigma }}_{u}^{2}/({\hat{\sigma }}_{u}^{2}+\sigma_{v}^{2})$. Likewise, we estimated the relative contributions of the spatially structured coefficients. For, say, $u_{i}^{wat}$, ${\hat{\sigma }}_{u_{i}^{wat}}^{2}=\sum_{i}\left(u_{i}^{wat}-{\bar{u}}^{wat}\right)/\left(n-1\right)$ is the empirical estimate for $\sigma_{u^{\left(\cdot \right)}}^{2}$. The proportion of variation for $u_{i}^{wat}$ is thus $frac_{u^{wat}}={\hat{\sigma }}_{u_{i}^{wat}}^{2}/\left({\hat{\sigma }}_{u_{i}^{wat}}^{2}+{\hat{\sigma }}_{u_{i}^{toi}}^{2}+{\hat{\sigma }}_{u_{i}^{disp}}^{2}\right)$. Our additional interest is on the uncertainties associated with the posterior means of the risk $\varsigma_{it}=\exp\left(\eta_{it}\right)$. Thus, the probability that the risk exceeds a threshold is $\varsigma_{it}^{Th}$. We estimated $\mathrm{Pr}(\varsigma_{it}>\varsigma^{Th})$, where $\varsigma^{Th}$ was set as the third quartile of the risk distribution. We fitted the model using the R-INLA package [29] together with the R software (R Core Team, Vienna, Austria) [36].

## 5. Results

**Figure 2:**
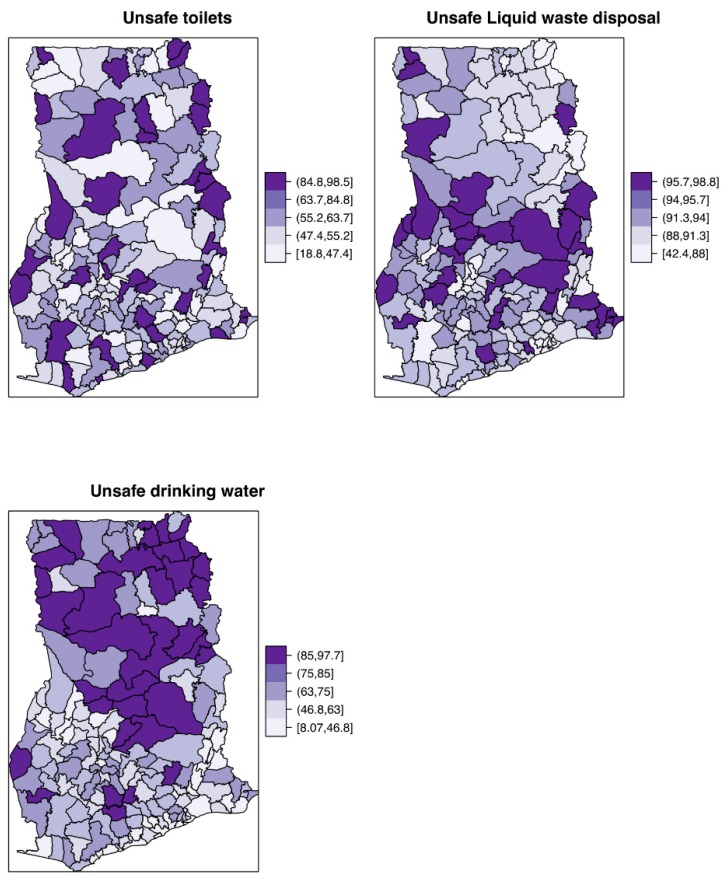
Spatial distribution of the constructed demographic covariates for proportions with unsafe toilets, unsafe drinking water, and unsafe liquid waste disposal.

Figure 2 shows the mapped distribution of the covariates, confirming variation in these explanatory factors. The proportion of the population with unsafe toilets, unsafe drinking water, and unsafe liquid waste disposal ranged from ≈ 19% to 98%, ≈ 8% to 98%, ≈ 42% to 99%, respectively. The Poisson-gamma model had a lower DIC value (DIC=9880.432) than the Poisson model (DIC=9886.555), hence it is preferred over the latter.

**Figure 3:**
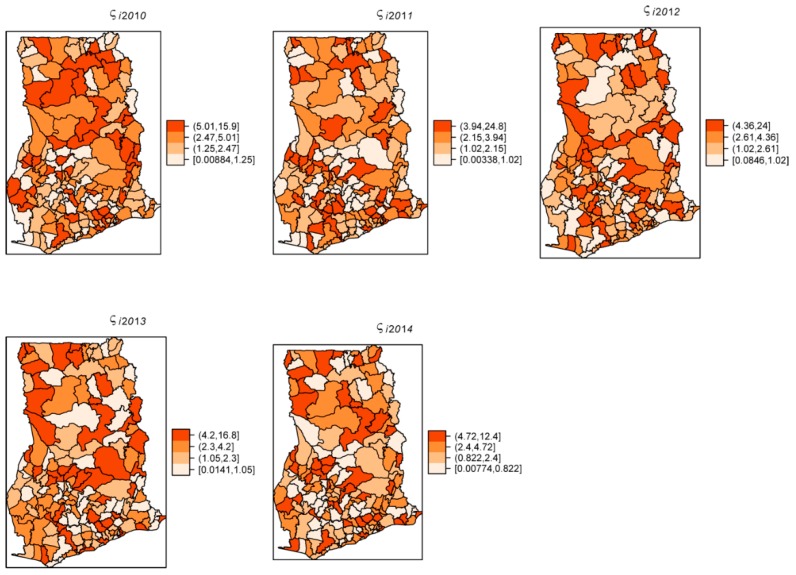
Yearly posterior risk estimates per 100 people of intestinal parasites infection, 2011–2014.

**Figure 4:**
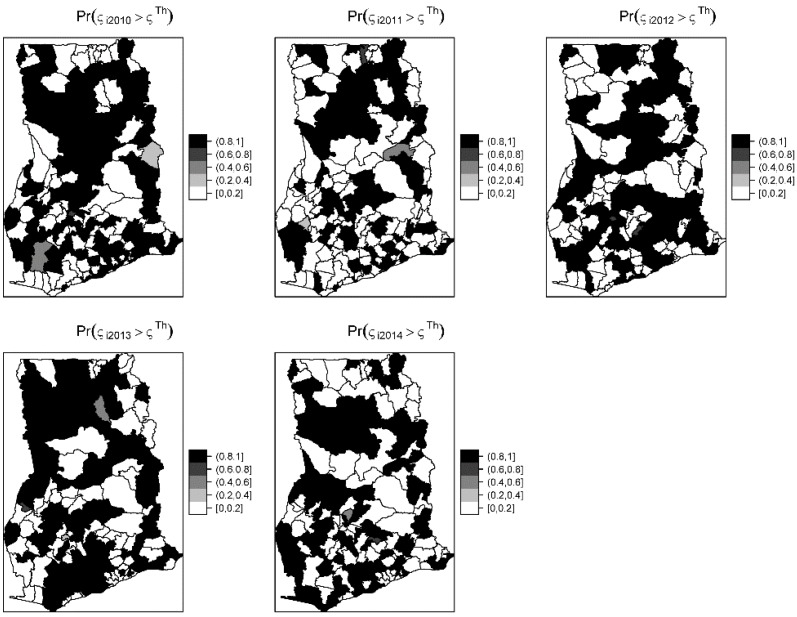
Yearly exceedance probability maps showing the probability that the posterior risk estimates exceed the 75th percentile or upper quartile threshold of the distribution.

**Figure 5:**
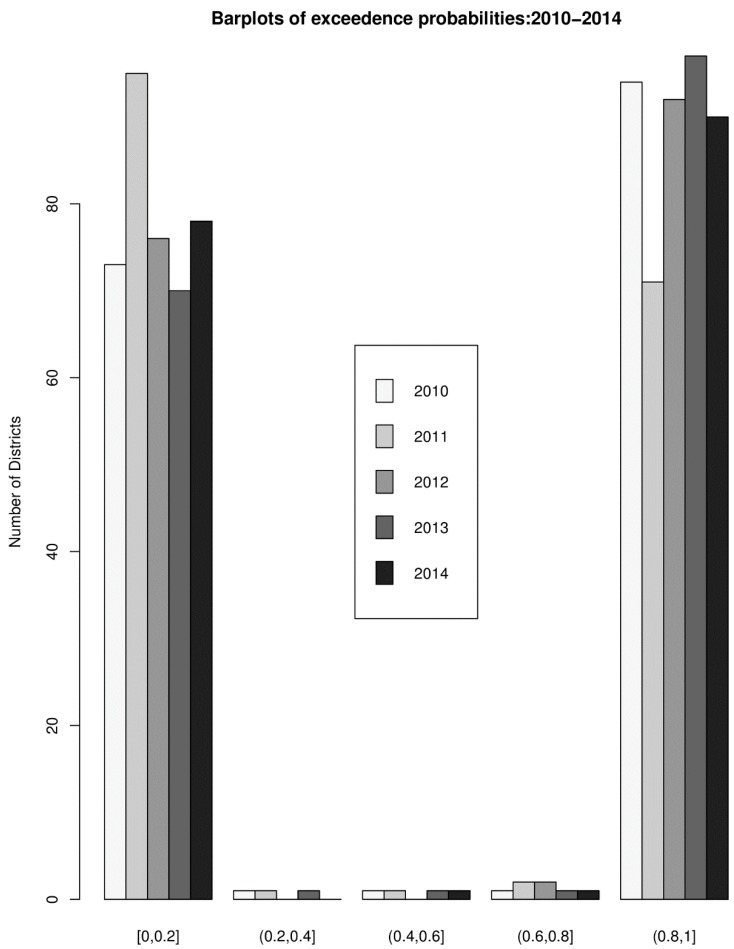
Bar plots of the number districts under each probability class for each year.

The overall risk of intestinal parasites infection was estimated to be 10.9 per 100 people. The mapped posterior estimates of intestinal parasites risk indicate spatial clustering of high and low risk, but it was patchier and more fragmented due to localized clustering (Figure 3). Figure 4 shows the exceedance probability maps indicating the probability of the posterior risks exceeding a threshold $\mathrm{Pr}(\varsigma_{it}>\varsigma^{Th})$. The areas with darker grey have at least 80% probability that their risk is greater than the threshold. Following Richardson et al. [37], we are confident to deduce that a high proportion of districts has a high probability of risk exceeding the upper quartile. Analysis of the exceedance probability maps is complemented by bar plots of the number districts under each probability class for each year (Figure 5). The number of districts with at least 80% probability of exceeding the threshold was in all cases higher than those below the threshold, except for the year 2011.

**Figure 6:**
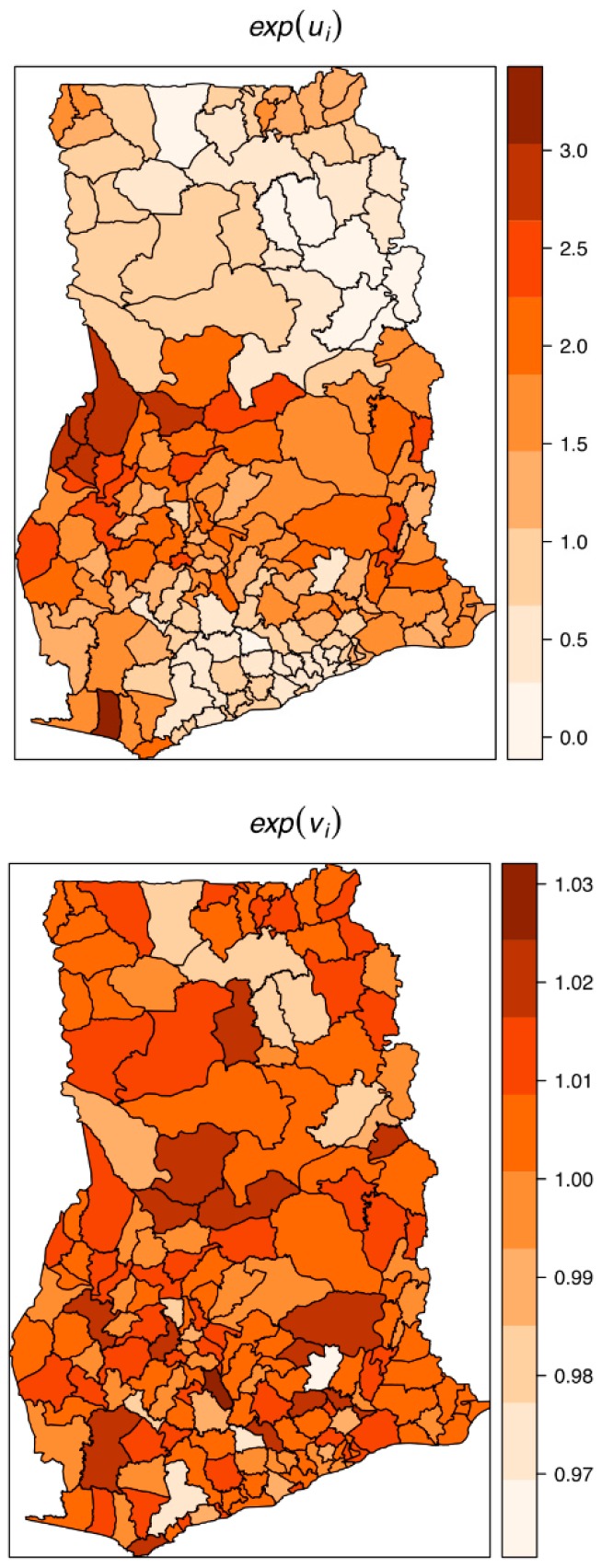
Residual spatial variation of the structured or clustering (top) and the unstructured or heterogeneity (below) after accounting for relevant covariates, temporal effects, and space-time effects.

**Figure 7:**
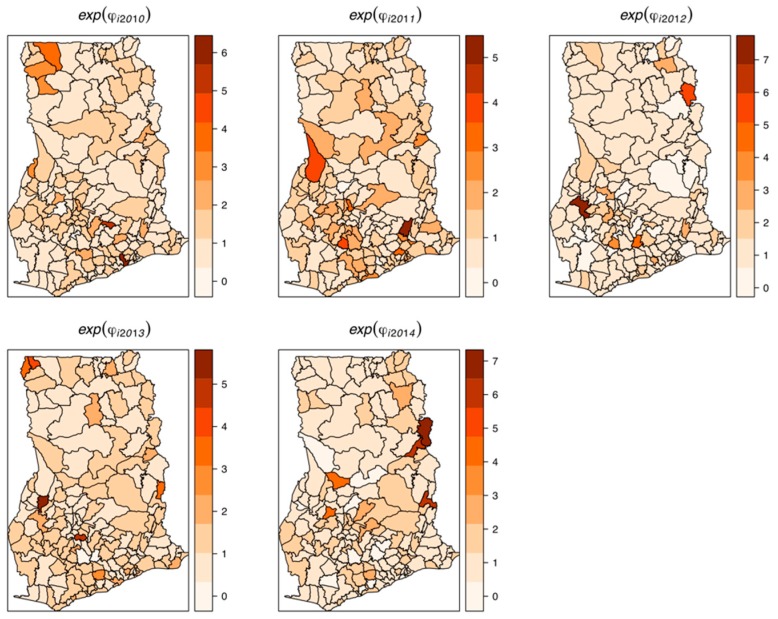
Maps of random space-time interaction effects, 2011–2014. These are also residual space-time variations after accounting for relevant covariates, temporal effects, and spatial effects.

**Figure 8:**
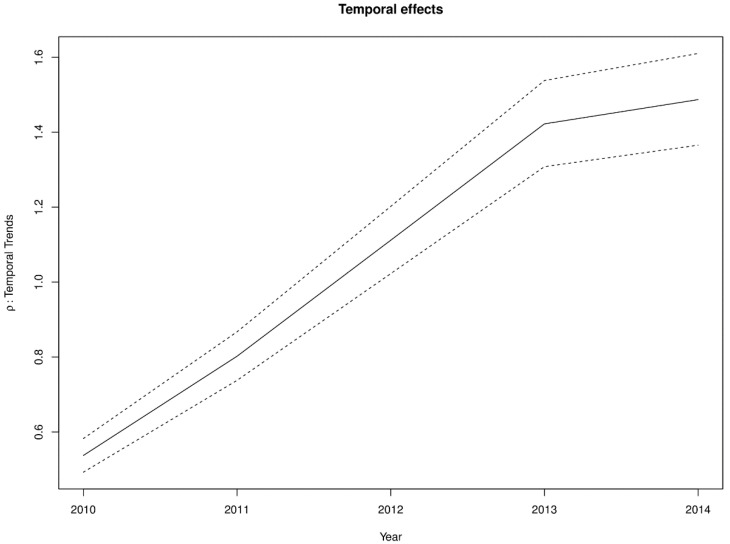
First-order random walk patterns of intestinal parasites risk from 2011–2014.

The estimated spatially structured variation (${\hat{\sigma }}_{u}^{2}=0.668$) widely dominated over the unstructured variation ($\sigma_{v}^{2}=0.0142$), explaining nearly 98% of the spatial random effects. This demonstrates a high level of spatial autocorrelation after accounting for model covariates. Figure 6 shows the spatial patterns of the residual spatial variation after accounting for model covariates, temporal effects, and spatio-temporal effects. The spatially structured component of the residual spatial variation widely varied with high-risk clusters of intestinal parasites infection within the middle belt of Ghana and low-risk clusters within the northern parts. The unstructured component showed marginal variation. Figure 7 shows the results of the space-time interaction effects. These are residual effects after accounting for covariates, temporal effects, and residual spatial effects. There is a consistent spatial pattern of this effect with a few isolated instances of divergence; thus, a few areas showed elevated values. Here, the areas with elevated values represent sporadic outbreaks or short-term clusters of intestinal parasites. Figure 8 shows the posterior random-walk effects on the exponential scale. There is a monotonic increase the expected risk with time, characterized by a steeper rate from 2010 to 2013, but less steep from 2013 to 2014.

**Figure 9:**
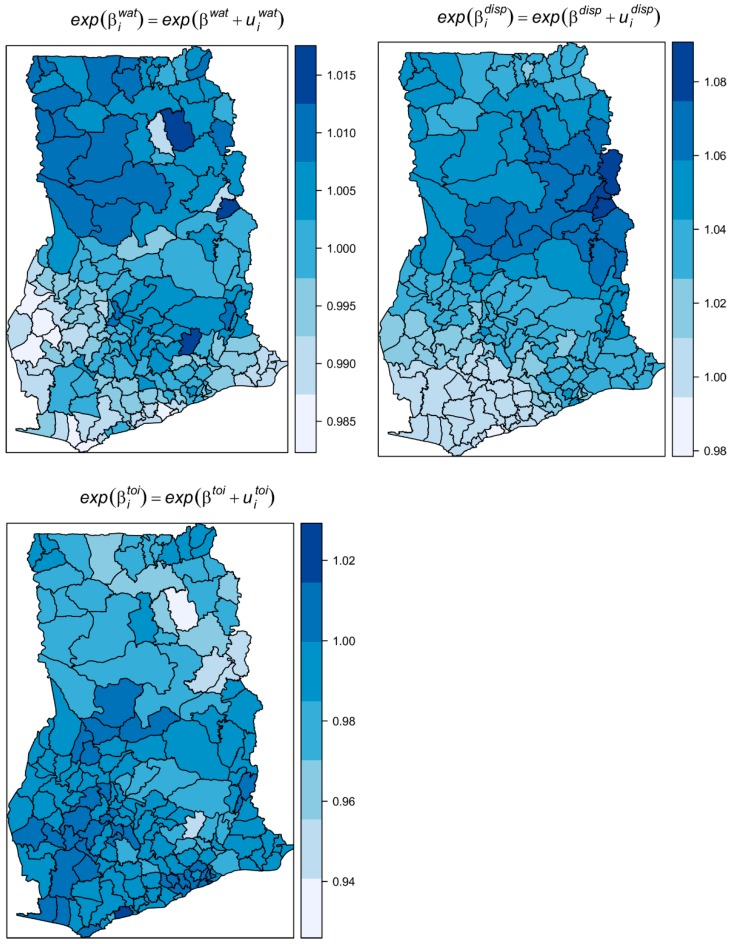
Maps of the spatially varying coefficients of unsafe water, unsafe toilets, and unsafe liquid waste disposal.

Table 1 shows the results of the fixed effects on the exponential scale and interpreted as multiplicative effects. The average expected estimated increase of infection was 3.2% for a 1% percent decrease in access to safe liquid waste disposal. Unexpectedly, the average expected increase in the risk was 1% for a percentage increase in the proportion of people with access to safe water. No substantial average change in risk was observed for a change in the proportion of unsafe toilets. The spatially varying coefficients $\beta_{i}^{wat}$, $\beta_{i}^{toi}$, and $\beta_{i}^{disp}$ show diverged changes in the risk with unit changes in $wat_{i}$, $toi_{i}$, and $disp_{i}$, respectively (Figure 9). Specifically, there were increasing effects in risk with increases in the proportion of unsafe drinking water, unsafe toilets, and unsafe liquid waste disposal, except for a few districts (light-colored areas) which showed no or contrasting effects. This finding suggests these districts have risk factors other than $wat_{i}$, $toi_{i}$, and $disp_{i}$. The contrasting effects were greater for $wat_{i}$; there were 110 out the 170 districts with contrasting effects for water. The $disp_{i}$ shows the covarite with the least contrasting effect (16 out 170 districts) followed by $toi_{i}$ (48 out 170). This also accords with the proportion of variation captured by each covariate. The variation captured by $\beta_{i}^{disp}$ dominated amongst the varying coefficients, capturing 64.7% of the total random slopes, followed by $\beta_{i}^{wat}$ (22.8%) and $\beta_{i}^{toi}$ (12.5%).

## 6. Discussion

This study presents the use of a spatially varying coefficient model to associate the spatial variation of intestinal parasites infection with demographic factors. Our study combines ideas from a Bayesian hierarchical framework with spatial, temporal, and space-time effects within the INLA modeling framework to evaluate the spatially varying effects of important demographic factors of intestinal parasites infection. Unlike global regression approaches which have often been used in previous studies, e.g., References [17,38,39,40], the model presented in this study has the advantage of estimating spatially varying effects of risk factors. These alleviate common challenges that could lead to wrong conclusions in epidemiological research. We identified the Poisson-gamma mixture likelihood distribution as being an appropriate one in this context as it adequately accounted for over-dispersion.

The risk maps indicate that the distribution of intestinal parasites is uneven across districts and is spatially clustered with many patches of high and low risks. The spatial patterns of the risk were patchier, probably because of the shorter scale of spatial dependency of the covariates and/or the varying shapes and sizes of the districts. Patchy risk areas could indicate resilience to high range disease transmission mechanisms, a hope for future interventions. The probability maps indicate that majority of the districts have a high probability of risk estimates exceeding the upper quartile. From the point of view of policymaking, monitoring, and control, the probability maps could aid the detection of areas characterized by extreme risk. The risk maps, complemented with the probability maps, could play a vital role as an important public health tool to guide monitoring and intervention programs.

The space-time interaction effects were high, with the isolated areas of elevated risk representing areas of short-term outbreaks needing interventions and further research. The residual spatial effects compensated for other demographic and environmental covariates and other socio-economic activities that were not captured in our study. The dominance of the structured residual spatial variation over the unstructured variation suggests that the possible unknown factors are spatially continuous in nature, and cluster at a larger spatial scale or same spatial scale of our study units.

The significant monotonic increase in the risk over time, as indicated by the random-walk effects, questions the effectiveness of public health monitoring and control of intestinal parasites infection in Ghana. The last two years of the study period is an exception, as even though there was an increase, but not at the same rate as previous years. This trend may parallel with current trends of population increases that have outstripped the provision of basic necessities like clean water and sanitation. This observation is not unique to Ghana. In sub-Saharan Africa in general, progress in reducing the prevalence has slowed compared to other regions of the world [1,2].

Our findings revealed unsafe water, unsafe toilets, and unsafe liquid waste disposal as important demographic factors that affect the risk of intestinal parasites infection. We observed that the effects of these factors vary substantially across districts, probably because of different dynamics of local interaction with these factors. From the global mean parameters, it is clear that fitting a global mean model would have obscured important characteristics of the effects. For instance, we observed a 3.2% global increase in the risk of infection by a unit increase in the proportion of the population with unsafe liquid waste disposal. Thus, a global mean model would partially explain the relationships, though substantial spatial variation occurs. There was up to an 8% increase in the risk within the middle belt and up to a 6% increase within the northern. Similarly, there are clear indications of varying effects for unsafe drinking water and unsafe toilets. The global mean effect suggests that unsafe toilet had no influence on the risk, yet there were considerable spatially varying effects. We expected a high proportion of unsafe drinking water to have a global increase in the risk in accordance with observations elsewhere [22]. Our findings showed otherwise and would have strange implications without knowledge of the varying effects. This reaffirms the significance of fitting varying coefficient models rather than global mean models, which have a high potential to obscure important covariate effects.

Our observation of the effects of unsafe liquid waste disposal to increase the risk of intestinal parasites infection accords with the nature of environments created when liquid waste is often disposed of within neighborhoods. Within our study area, limited access to safe liquid waste disposal implies that inhabitants mostly dispose of their liquid waste in the compound of neighborhoods. This can create an adequate soil moisture content to enhance development and survival of the ova and larvae of parasites, easy dissemination of parasites, and increase of the ability for mobile larvae to migrate to more moisture conditions when avoiding desiccation [4,5]. *Ascaris lumbricoides* and *Trichuris trichiura* eggs have been known to develop [41] and survive in soils with adequate moisture contents [42]. Using vegetation as a proxy for adequate soil moisture, since it provides shade and protects eggs from ultraviolet radiation and desiccation, Manz et al. [43] associated the survival of *Ascaris lumbricoides* and *Trichuris trichiura* eggs with soil moisture. We found no substantial effects for unsafe liquid waste disposal for districts within the southwestern parts, therefore justifying the need for a further epidemiological investigation.

The observed increases in the risk with unsafe water and unsafe toilets for most districts were within our expectation. Insufficient access to safe toilets and drinking water are intimately connected [44], the combination of which is a stark indicator of poor hygienic and living conditions, which are mostly attributed to increasing the risk of infection [22]. The transmission of most intestinal parasites occurs via contaminated food or water (fecal–oral route), infected fingers, or through the skin by walking on larvae-infected soil [45], which could be accelerated by an unsafe toilet and unsafe water. Inhabitants lacking access to household sanitation have an increased risk of water or food contamination, hence a higher risk of infection [46]. Limited availability of safe drinking water implies that inhabitants resort to unprotected wells and rivers, shallow waters, and tanker supplies, which are highly susceptible to fecal contamination. Drinking water from such sources has been associated with a high risk of intestinal parasites amongst school children elsewhere [47]. Districts with higher proportions of unsafe toilets have an elevated risk of contamination with human excreta as are those with high proportions of unsafe drinking water, thereby enhancing fecal–oral transmissions. In fact, inhabitants without access to toilets resort to indiscriminate defecation in the environment or open fields, hence could increase the risk of human–parasites interaction and infection [45,48].

Several districts showed effects of unsafe water, unsafe toilet, and unsafe liquid waste disposal that contrasted our expectation, and require further epidemiological research. This is pronounced within the southwestern parts for unsafe toilet and the northern parts for unsafe water, suggesting that these factors are poor predictors of intestinal parasites risk in these regions. Environmental conditions such as soil type and temperature may explain these observed effects. This part of Ghana is characterized by extreme temperature and dry soil conditions, which have been associated with the absence of intestinal parasites like hookworm infection [22].

The study has some limitations. The data were lumped as intestinal parasites morbidities without considering separate analyses for hookworm or ringworm morbidities. The study was conducted within an ecological regression modeling framework without individual-level covariates. Therefore, our inferences are based on a group level rather than individual levels. Better measures, however, will be included in the future.

## 7. Conclusions

We conclude the following strengths of this study. First, the study adds to the current literature on the epidemiology of intestinal parasites infection modeling with a hierarchical Bayesian framework. Most spatial epidemiological studies of intestinal parasites rely on cross-sectional survey data that are expensive to collect and difficult to update within short periods. The use of morbidity data, as in our case, provides opportunities to make timely updates of incidences over large areas, even for a whole country. Second, the study has evaluated the spatially varying effects of demographic factors on the spatial variation of intestinal parasites infection. This approach of ecological modeling is rarely applied in the spatial epidemiology of intestinal parasites infection. The spatially varying coefficient model unveiled important dependencies, which would otherwise have been obscured by global mean models. Our findings noted unsafe drinking water, unsafe toilets, and unsafe liquid waste disposal are important demographic factors that account for the spatial variation of intestinal parasites infections. Finally, the results of this study have vital implications in aiding public health monitoring and control of intestinal parasites in Ghana as areas of high probabilities of excess risk could be easily identified for further attention.

## Figures and Tables

**Table 1:** Parameter estimates of the Poison-gamma mixture model.

Parameters	Estimates (95% Confidence Interval)
$exp\left(\beta_{0}\right)$	0.019 (0.015–0.024)
**Continuous**	
$exp\left(\beta^{wat}\right)$	0.990 (0.980–1.005)
$exp\left(\beta^{toi}\right)$	0.999 (0.997–1.068)
$exp\left(\beta^{disp}\right)$	1.032 (0.998–1.257)
**% variance**	
Residual risk variation	
$v_{i}$	2.6
$u_{i}$	98.4
Residual covariates effects	
$u_{i}^{wat}$	22.8
$u_{i}^{toi}$	12.5
$u_{i}^{disp}$	64.7
**No. of contrasting districts**	
$u_{i}^{wat}$	110
$u_{i}^{toi}$	48
$u_{i}^{disp}$	16

## References

[1] Bethony J., Brooker S., Albonico M., Geiger S.M., Loukas A., Diemert D., Hotez P.J. (2006). Soil-transmitted helminth infections: Ascariasis, trichuriasis, and hookworm. Lancet.

[2] De Silva N.R., Brooker S., Hotez P.J., Montresor A., Engels D., Savioli L. (2003). Soil-transmitted helminth infections: Updating the global picture. Trends Parasitol..

[3] Alum A., Rubino J.R., Ijaz M.K. (2010). The global war against intestinal parasites—Should we use a holistic approach?. Int. J. Infect. Dis..

[4] Brooker S., Clements A.C., Bundy D.A. (2006). Global epidemiology, ecology and control of soil-transmitted helminth infections. Adv. Parasitol..

[5] Brooker S., Michael E. (2000). The potential of geographical information systems and remote sensing in the epidemiology and control of human helminth infections. Adv. Parasitol..

[6] Chammartin F., Guimarães L.H., Scholte R.G., Bavia M.E., Utzinger J., Vounatsou P. (2014). Spatio-temporal distribution of soil-transmitted helminth infections in Brazil. Parasites Vectors.

[7] Stephenson L.S., Latham M.C., Adams E.J., Kinoti S.N., Pertet A. (1993). Physical fitness, growth and appetite of Kenyan school boys with hookworm, Trichuris trichiura and Ascaris lumbricoides infections are improved four months after a single dose of albendazole. J. Nutr..

[8] Stephenson L.S., Latham M.C., Kinoti S.N., Kurz K.M., Brigham H. (1990). Improvements in physical fitness of Kenyan schoolboys infected with hookworm, *Trichuris trichiura* and *Ascaris lumbricoides* following a single dose of albendazole. Trans. R. Soc. Trop. Med. Hyg..

[9] Stephenson L.S., Latham M.C., Kurz K.M., Kinoti S.N., Brigham H. (1989). Treatment with a single dose of albendazole improves growth of Kenyan schoolchildren with hookworm, Trichuris trichiura, and Ascaris lumbricoides infections. Am. J. Trop. Med. Hyg..

[10] Annan A., Crompton D.W., Walters D.E., Arnold S.E. (1986). An investigation of the prevalence of intestinal parasites in pre-school children in Ghana. Parasitology.

[11] Adjei A.A., Armah H., Rodrigues O., Renner L., Borketey P., Ayeh-Kumi P., Adiku T., Sifah E., Lartey M. (2004). *Cryptosporidium* Spp., a frequent cause of diarrhea among children at the Korle-Bu Teaching Hospital, Accra, Ghana. Jpn. J. Infect. Dis..

[12] Baidoo S.E., Tay S.C.K., Abruquah H.H. (2010). Intestinal helminth infection and anaemia during pregnancy: A community based study in Ghana. Afr. J. Microbiol. Res..

[13] Duedu K., Peprah E., Anim-Baidoo I., Ayeh-Kumi P.F. (2015). Prevalence of Intestinal Parasites and Association with Malnutrition at a Ghanaian Orphanage. Hum. Parasit. Dis..

[14] Duedu K.O., Karikari Y.A., Attah S.K., Ayeh-Kumi P.F. (2015). Prevalence of intestinal parasites among patients of a Ghanaian psychiatry hospital. BMC Res. Notes.

[15] Humphries D., Simms B.T., Davey D., Otchere J., Quagraine J., Terryah S., Newton S., Berg E., Harrison L.M., Boakye D. (2013). Hookworm Infection among School Age Children in Kintampo North Municipality, Ghana: Nutritional Risk Factors and Response to Albendazole Treatment. Am. J. Trop. Med. Hyg..

[16] Ayeh-Kumi P.F., Quarcoo S., Kwakye-Nuako G., Kretchy J.P., Osafo Kantanka A., Mortu S. (2009). Prevalence of intestinal parasitic infections among food vendors in Accra, Ghana. J. Trop. Med. Parasitol..

[17] Magalhães R.J.S., Biritwum N.-K., Gyapong J.O., Brooker S., Zhang Y., Blair L., Fenwick A., Clements A.C.A. (2011). Mapping Helminth Co-Infection and Co-Intensity: Geostatistical Prediction in Ghana. PLoS Negl. Trop. Dis..

[18] Kabore A., Biritwum N.-K., Downs P.W., Magalhaes R.J.S., Zhang Y., Ottesen E.A. (2013). Predictive vs. Empiric Assessment of Schistosomiasis: Implications for Treatment Projections in Ghana. PLoS Negl. Trop. Dis..

[19] World Health Organization (2012). Soil-Transmitted Halminthiases: STH: Eliminating Soil-Transmitted Helminthiases as a Public Health Problem in Children: Progress Report 2001–2010 and Strategic Plan 2011–2020.

[20] Brooker S., Clements A.C.A. (2009). Spatial heterogeneity of parasite co-infection: Determinants and geostatistical prediction at regional scales. Int. J. Parasitol..

[21] Fuhrimann S., Winkler M.S., Kabatereine N.B., Tukahebwa E.M., Halage A.A., Rutebemberwa E., Medlicott K., Schindler C., Utzinger J., Cissé G. (2016). Risk of Intestinal Parasitic Infections in People with Different Exposures to Wastewater and Fecal Sludge in Kampala, Uganda: A Cross-Sectional Study. PLoS Negl. Trop. Dis..

[22] Karagiannis-Voules D.-A., Biedermann P., Ekpo U.F., Garba A., Langer E., Mathieu E., Midzi N., Mwinzi P., Polderman A.M., Raso G. (2015). Spatial and temporal distribution of soil-transmitted helminth infection in sub-Saharan Africa: A systematic review and geostatistical meta-analysis. Lancet Infect. Dis..

[23] Nobre L.N., Silva R.V., Macedo M.S., Teixeira R.A., Lamounier J.A., Franceschini S.C.C. (2013). Risk factors for intestinal parasitic infections in preschoolers in a low socio-economic area, Diamantina, Brazil. Pathog. Glob. Health.

[24] Fuhrimann S., Winkler M.S., Pham-Duc P., Do-Trung D., Schindler C., Utzinger J., Cissé G. (2016). Intestinal parasite infections and associated risk factors in communities exposed to wastewater in urban and peri-urban transition zones in Hanoi, Vietnam. Parasites Vectors.

[25] Turki H., Hamedi Y., Heidari-Hengami M., Najafi-Asl M., Rafati S., Sharifi-Sarasiabi K. (2017). Prevalence of intestinal parasitic infection among primary school children in southern Iran. J. Parasit. Dis..

[26] Matthys B., Bobieva M., Karimova G., Mengliboeva Z., Jean-Richard V., Hoimnazarova M., Kurbonova M., Lohourignon L.K., Utzinger J., Wyss K. (2011). Prevalence and risk factors of helminths and intestinal protozoa infections among children from primary schools in western Tajikistan. Parasites Vectors.

[27] Osei F.B., Stein A. (2017). Spatio-temporal analysis of small-area intestinal parasites infections in Ghana. Sci. Rep..

[28] Rue H., Martino S. (2007). Approximate Bayesian inference for hierarchical Gaussian Markov random field models. J. Stat. Plan. Inference.

[29] Martins T.G., Simpson D., Lindgren F., Rue H. (2013). Bayesian computing with INLA: New features. Comput. Stat. Data Anal..

[30] Gyimah S.O. Interaction Effects of Maternal Education and Household Facilities on Childhood Diarrhea in Sub-Saharan Africa: The Case of Ghana. http://www.longwoods.com/content/17628.

[31] Awuah E., Nyarko K.B., Owusu P.A. (2009). Water and sanitation in Ghana. Desalination.

[32] Osei F.B., Stein A. (2017). Diarrhea Morbidities in Small Areas: Accounting for Non-Stationarity in Sociodemographic Impacts using Bayesian Spatially Varying Coefficient Modelling. Sci. Rep..

[33] Besag J., York J., Mollié A. (1991). Bayesian image restoration, with two applications in spatial statistics. Ann. Inst. Stat. Math..

[34] Knorr-Held L. (2000). Bayesian modelling of inseparable space-time variation in disease risk. Stat. Med..

[35] Spiegelhalter D.J., Best N.G., Carlin B.P., Van Der Linde A. (2002). Bayesian measures of model complexity and fit. J. R. Stat. Soc. Ser. B.

[36] R Core Team (2016). R: A Language and Environment for Statistical Computing.

[37] Richardson S., Thomson A., Best N., Elliott P. (2004). Interpreting Posterior Relative Risk Estimates in Disease-Mapping Studies. Environ. Health Perspect..

[38] Magalhães R.J.S., Salamat M.S., Leonardo L., Gray D.J., Carabin H., Halton K., McManus D.P., Williams G.M., Rivera P., Saniel O. (2015). Mapping the Risk of Soil-Transmitted Helminthic Infections in the Philippines. PLoS Negl. Trop. Dis..

[39] Yapi R.B., Chammartin F., Hürlimann E., Houngbedji C.A., N’Dri P.B., Silué K.D., Utzinger J., N’Goran E.K., Vounatsou P., Raso G. (2016). Bayesian risk profiling of soil-transmitted helminth infections and estimates of preventive chemotherapy for school-aged children in Côte d’Ivoire. Parasites Vectors.

[40] Chammartin F., Scholte R.G., Malone J.B., Bavia M.E., Nieto P., Utzinger J., Vounatsou P. (2013). Modelling the geographical distribution of soil-transmitted helminth infections in Bolivia. Parasites Vectors.

[41] Baron S. (1996). Medical Microbiology.

[42] Spindler L.A. (1929). The Relation oî Moisture to the Distribution of Human Trichuris and Ascaris. Am. J. Hyg..

[43] Manz K.M., Clowes P., Kroidl I., Kowuor D.O., Geldmacher C., Ntinginya N.E., Maboko L., Hoelscher M., Saathoff E. (2017). Trichuris trichiura infection and its relation to environmental factors in Mbeya region, Tanzania: A cross-sectional, population-based study. PLoS ONE.

[44] Curtis V., Kanki B., Mertens T. (1995). Pots. pits and pipes: Explaining hygiene behaviour in burkina faso. Soc. Sci. Med..

[45] Kattula D., Sarkar R., Ajjampur S.S.R., Minz S., Levecke B., Muliyil J., Kang G. (2014). Prevalence & risk factors for soil transmitted helminth infection among school children in south India. Indian J. Med. Res..

[46] McKenna M.L., McAtee S., Bryan P.E., Jeun R., Ward T., Kraus J., Bottazzi M.E., Hotez P.J., Flowers C.C., Mejia R. (2017). Human Intestinal Parasite Burden and Poor Sanitation in Rural Alabama. Am. J. Trop. Med. Hyg..

[47] Grimes J.E.T., Tadesse G., Mekete K., Wuletaw Y., Gebretsadik A., French M.D., Harrison W.E., Drake L.J., Gardiner I.A., Yard E. (2016). School Water, Sanitation, and Hygiene, Soil-Transmitted Helminths, and Schistosomes: National Mapping in Ethiopia. PLoS Negl. Trop. Dis..

[48] Khan M.Y. (1979). An analytical study of factors related to infestation by intestinal parasites in rural school children (report of a pilot study). Public Health.

